# Supplementary material for: Emotional, Behavioral, and Physical Health Consequences in Caregivers of Children with Cancer: A Network Analysis Differentiation in Mothers’ and Fathers’ Reactivity
Source: Cancers (Basel). 2023 Jul 4;15(13):3496. doi: 10.3390/cancers15133496 (PMC10340596; doi:10.3390/cancers15133496)
Supplement: Supplementary file 1 [file cancers-15-03496-s001.zip › Supplementary S1.pdf]

1. Kellner's Symptom Questionnaire

Please describe how you have felt DURING THE PAST WEEK TO DAY and make a small check mark like this √ .
For example the word NERVOUS is on the first line: if you have felt nervous check YES like this √YES NO
If you have not felt nervous check NO like this YES √NO
A few times you have the choice of checking either TRUE or FALSE.
Do not think long before answering. Work quickly!

1	Nervous	YES	NO
2	Weary	YES	NO
3	Irritable	YES	NO
4	Cheerful	YES	NO
5	Tense, tensed up	YES	NO
6	Sad, blue	YES	NO
7	Happy	YES	NO
8	Frightened	YES	NO
9	Feeling Calm	YES	NO
10	Feeling healthy	YES	NO
11	Loosing temper easily	YES	NO
12	Feeling of not enough air	TRUE	FALSE
13	Feeling kind toward people	YES	NO
14	Feeling fit	YES	NO
15	Heavy arms or legs	YES	NO
16	Feeling confident	YES	NO
17	Feeling warm toward people	YES	NO
18	Shaky	YES	NO
19	No pain anywhere	TRUE	FALSE
20	Angry	YES	NO
21	Arms and legs feel strong	YES	NO
22	Appetite poor	YES	NO
23	Feeling peaceful	YES	NO
24	Feeling unworthy	YES	NO
25	Annoyed	YES	NO
26	Feeling of rage	YES	NO
27	Cannot enjoy yourself	YES	NO
28	Tight head or neck	YES	NO
29	Relaxed	YES	NO
30	Restless	YES	NO
31	Feeling friendly	YES	NO
32	Feeling of hate	YES	NO
33	Choking feeling	YES	NO
34	Afraid	YES	NO
35	Patient	YES	NO
36	Scared	YES	NO
37	Furious	YES	NO
38	Feeling charitable, forgiving	YES	NO
39	Feeling guilty	YES	NO
40	Feeing well	YES	NO
41	Feeling of pressure in head or body	YES	NO
42	Worried	YES	NO
43	Contented	YES	NO
44	Weak arms or legs	YES	NO
45	Feeling desperate, terrible	YES	NO

46	No aches anywhere	TRUE	FALSE
47	Thinking of death or dying	YES	NO
48	Hot tempered	YES	NO
49	Terrified	YES	NO
50	Feeling of courage	YES	NO
51	Enjoying yourself	YES	NO
52	Breathing difficult	YES	NO
53	Parts of the body feel numb or tingling	YES	NO
54	Takes a long time to fall asleep	YES	NO
55	Feeling hostile	YES	NO
56	Infuriated	YES	NO
57	Heart beating fast or pounding	YES	NO
58	Depressed	YES	NO
59	Jumpy	YES	NO
60	Feeling a failure	YES	NO
61	Not interested in things	TRUE	FALSE
62	Highly strung	YES	NO
63	Cannot relax	TRUE	FALSE
64	Panicky	YES	NO
65	Pressure on head	YES	NO
66	Blaming yourself	YES	NO
67	Thoughts of ending your life	YES	NO
68	Frightening thoughts	YES	NO
69	Enraged	YES	NO
70	Irritated by other people	YES	NO
71	Looking forward toward the future	YES	NO
72	Nauseated, sick to stomach	YES	NO
73	Feeling that life is bad	YES	NO
74	Upset bowels or stomach	YES	NO
75	Feeling inferior to others	YES	NO
76	Feeling useless	YES	NO
77	Muscle pains	YES	NO
78	No unpleasant feeling s in head or body	TRUE	FALSE
79	Headaches	YES	NO
80	Feel like attacking people	YES	NO
81	Shaking with anger	YES	NO
82	Mad	YES	NO
83	Feeling goodwill	YES	NO
84	Feel like crying	YES	NO
85	Cramps	YES	NO
86	Feeling that something bad will happen	YES	NO
87	Wound up, uptight	YES	NO
88	Get angry quickly	YES	NO
89	Self-confident	YES	NO
90	Resentful	YES	NO
91	Feeling of hopelessness	YES	NO
92	Head pains	YES	NO

Scoring Instructions:

For symptom subscales add together the following items and score 1 when the answer is Yes/True:

Anxiety (range 0-17): 1, 5, 8, 18, 30, 34, 36, 42, 49, 54, 59, 62,63,64, 68, 86,87

Depression (range 0-17): 2,6,24,27,39,45,47, 58,60,61,66,67,73,75,76,84,91

Somatic Symptoms(range 0-17):12,15,22,28,33,41,44,52,53,57,65,72,74,77,79,85,92

Hostility(range 0-17): 3,11,20,25,26,32,37,48,55,56,69,80,81,82,88,90

For well-being subscales add together the following items and score 1 when the answer is No/False:

Relaxation (range 0-6): 9,16,23,29,50,89

Contentment (range 0-6): 4,7,40,43,51,71

Physical Well-being (range 0-6):10,14,19,21,46,78

Friendliness (range 0-6): 13,17,31,35, 38,83

For the total scale score, add together the symptom subscale with each corresponding well-being subscale (see Table 1 in the text) :

Anxiety (range 0-23): Anxiety +Relaxation

Depression (range 0-23): Depression+Contentment

Somatization (range 0-23): Somatic Symptoms + Physical Well-Being

Hostility (range 0-23): Hostility + Friendliness
