# Supplementary figures and images for: Emotional, Behavioral, and Physical Health Consequences in Caregivers of Children with Cancer: A Network Analysis Differentiation in Mothers’ and Fathers’ Reactivity
Source: Cancers (Basel). 2023 Jul 4;15(13):3496. doi: 10.3390/cancers15133496 (PMC10340596; doi:10.3390/cancers15133496)

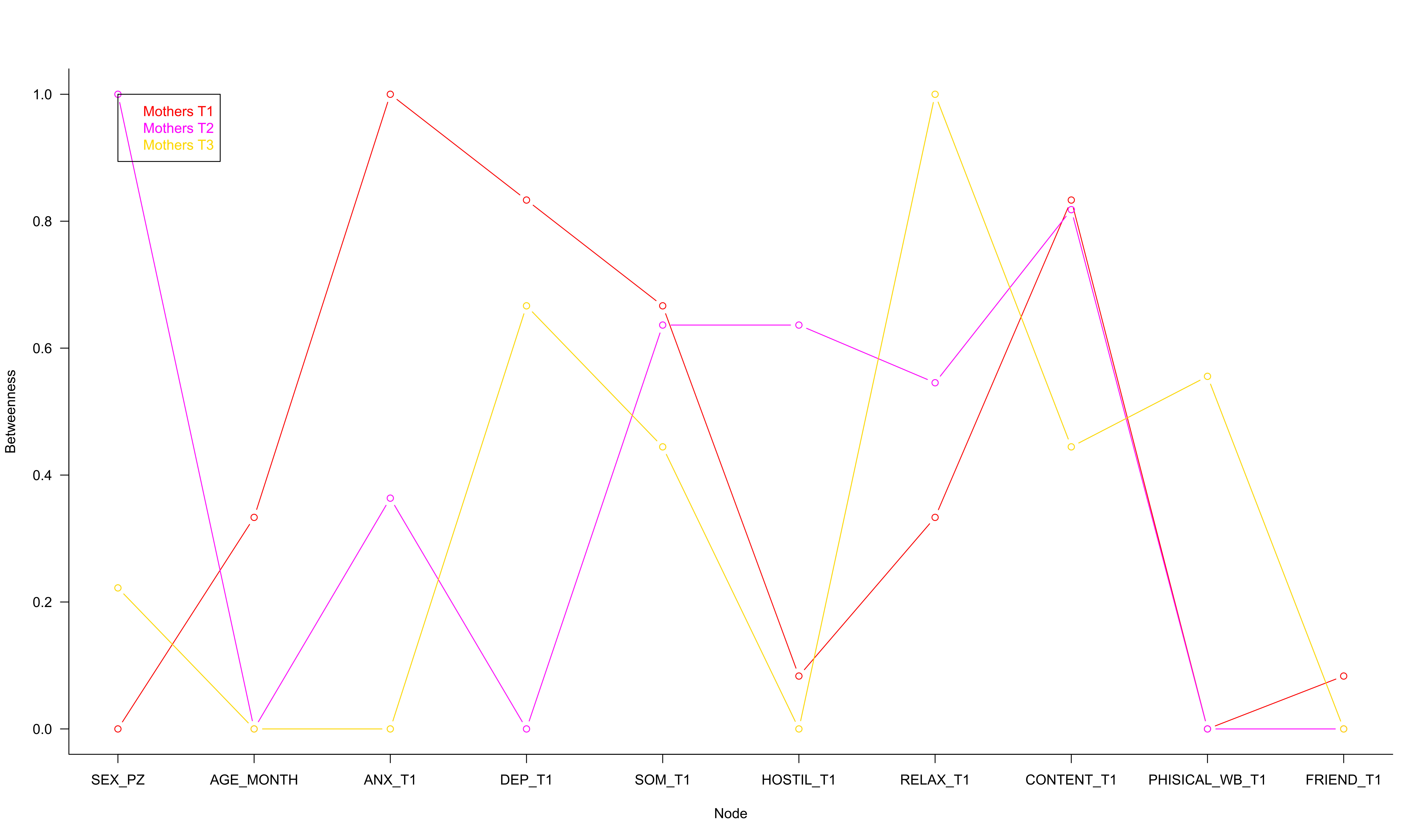

Supplement: Supplementary file 1 [file cancers-15-03496-s001.zip › Supplementary S2.jpeg]

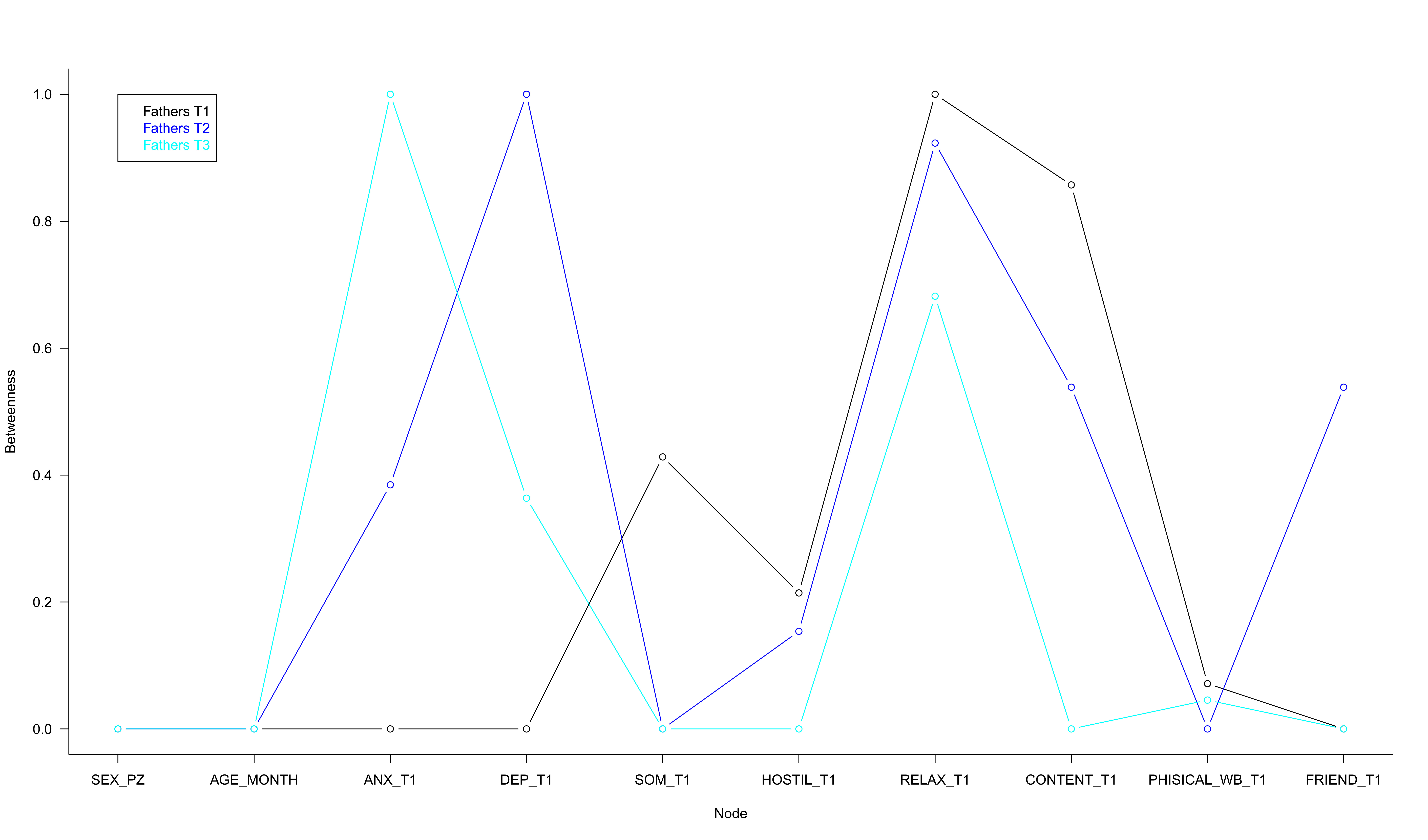

Supplement: Supplementary file 1 [file cancers-15-03496-s001.zip › Supplementary S3.jpeg]
